# Selective Translation of Low Abundance and Upregulated Transcripts in Halobacterium salinarum

**DOI:** 10.1128/mSystems.00329-20

**Published:** 2020-07-28

**Authors:** Adrián López García de Lomana, Ulrike Kusebauch, Arjun V. Raman, Min Pan, Serdar Turkarslan, Alan P. R. Lorenzetti, Robert L. Moritz, Nitin S. Baliga

**Affiliations:** a Institute for Systems Biology, Seattle, Washington, USA; b Microbial Systems Biology Lab, Universidade de São Paulo, Ribeirão Preto, Brazil; c Department of Biology, University of Washington, Seattle, Washington, USA; d Department of Microbiology, University of Washington, Seattle, Washington, USA; e Molecular and Cellular Biology Program, University of Washington, Seattle, Washington, USA; f Lawrence Berkeley National Lab, Berkeley, California, USA; Institute for Genomics & Systems Biology

**Keywords:** translational regulation, selective translation, transcription-translation interplay, ribosome heterogeneity, transcriptomics, ribosome profiling, proteomics, archaea

## Abstract

When organisms encounter an unfavorable environment, they transition to a physiologically distinct, quiescent state wherein abundant transcripts from the previous active growth state continue to persist, albeit their active transcription is downregulated. In order to generate proteins for the new quiescent physiological state, we hypothesized that the translation machinery must selectively translate upregulated transcripts in an intracellular milieu crowded with considerably higher abundance transcripts from the previous active growth state. Here, we have analyzed genome-wide changes in the transcriptome (RNA sequencing [RNA-seq]), changes in translational regulation and efficiency by ribosome profiling across all transcripts (ribosome profiling [Ribo-seq]), and protein level changes in assembled ribosomal proteins (sequential window acquisition of all theoretical mass spectra [SWATH-MS]) to investigate the interplay of transcriptional and translational regulation in Halobacterium salinarum as it transitions from active growth to quiescence. We have discovered that interplay of regulatory processes at different levels of information processing generates condition-specific ribosomal complexes to translate preferentially pools of low abundance and upregulated transcripts. Through analysis of the gene regulatory network architecture of H. salinarum, Escherichia coli, and Saccharomyces cerevisiae, we demonstrate that this conditional, modular organization of regulatory programs governing translational systems is a generalized feature across all domains of life.

**IMPORTANCE** Our findings demonstrate conclusively that low abundance and upregulated transcripts are preferentially translated, potentially by environment-specific translation systems with distinct ribosomal protein composition. We show that a complex interplay of transcriptional and posttranscriptional regulation underlies the conditional and modular regulatory programs that generate ribosomes of distinct protein composition. The modular regulation of ribosomal proteins with other transcription, translation, and metabolic genes is generalizable to bacterial and eukaryotic microbes. These findings are relevant to how microorganisms adapt to unfavorable environments when they transition from active growth to quiescence by generating proteins from upregulated transcripts that are in considerably lower abundance relative to transcripts associated with the previous physiological state. Selective translation of transcripts by distinct ribosomes could form the basis for adaptive evolution to new environments through a modular regulation of the translational systems.

## Abstract (precis)

Our findings demonstrate conclusively that low abundance and upregulated transcripts are preferentially translated, potentially by environment-specific translation systems with distinct ribosomal protein composition. We show that a complex interplay of transcriptional and posttranscriptional regulation underlies the conditional and modular regulatory programs that generate ribosomes of distinct protein composition. The modular regulation of ribosomal proteins with other transcription, translation, and metabolic genes is generalizable to bacterial and eukaryotic microbes. These findings are relevant to how microorganisms adapt to unfavorable environments when they transition from active growth to quiescence by generating proteins from upregulated transcripts that are in considerably lower abundance relative to transcripts associated with the previous physiological state. Selective translation of transcripts by distinct ribosomes could form the basis for adaptive evolution to new environments through a modular regulation of the translational systems.

## INTRODUCTION

The concept that functional heterogeneity results from variations in the translational machinery is gaining renewed support (1). Variations in ribosomal proteins (RPs), ribosomal RNAs (rRNAs), transfer RNAs (tRNAs), and translation factors are four molecular axes that provide support for translational regulation (2, 3). Translational regulation plays an important role in fundamental biological processes like vertebrate development, where ribosomes with specific subunit composition, i.e., presence or absence of ribosomal protein RPL10A/uL1 and RPS25/eS25, preferentially translate functionally distinct pools of mRNAs in murine stem cells (4). Also in vertebrate development, ribosome-mediated translational specificity occurs through direct interaction between RP L38 and structural RNA elements resembling internal ribosome entry sites in the 5′ untranslated region (UTR) of select Hox mRNAs (5, 6). Furthermore, selective translation modulates stress response and various complex human disease phenotypes (7, 8). Variations in RP stoichiometry with functional implications have been reported for Saccharomyces cerevisiae (9), such that RP Asc1/RACK1 is required for efficient translation of short mRNAs (10), and Rps26-depleted ribosomes support stress responses (11, 12). Moreover, a ribosome code has been proposed to explain the divergence in phenotypic outcome when individual paralogs of duplicated RP genes are deleted (13). Not only variations in RP stoichiometry (14) but also 16S rRNA variation in Escherichia coli has been associated with functional differences that can regulate stress response via RpoS and RelA regulons (15). While the ultimate functional consequences of ribosome diversity require careful experimental validation (16) and meticulous interpretation in light of alternative regulatory mechanisms (17), these studies argue against the notion that the ribosome is a rote translation machine, but rather a context-sensitive regulatory control element steering conditional protein synthesis (14, 18). Compared to the organisms above, fewer studies have been performed in the archaea domain. Nevertheless, translation initiation is suggested to utilize a completely novel mechanism in haloarchaea (19). In the archaeon Sulfolobus acidocaldarius, RP L7Ae binds to select coding and noncoding RNAs, including its own mRNA, to regulate translation (20).

In this article, we explore the interplay of translational and transcriptional regulation in driving microbial transitions to quiescent states upon encountering unfavorable environments. Upon exposure to a stressful environment, microorganisms elicit protective and acclimation responses that are often associated with a dormant or quiescent phenotypic state (21). We previously used a systems biology approach to investigate transcript and protein level changes in Halobacterium salinarum when this halophilic archaeon underwent transition from active to quiescent growth states in batch cultures (22) and in response to a controlled switch from favorable oxic to unfavorable anoxic conditions (23). We discovered that upon encountering nutrient depletion or anoxic conditions, a large fraction of genes (51% in batch cultures [24] and 9% in oxygen transitions [23]) in the genome were differentially regulated. Notably, the drop in ATP level partially explained why downregulated transcripts from the active growth state continued to persist in the stationary phase and under anoxic conditions. Two observations highlighted the differences in translational regulation across transitions between active growth and quiescent states. First, even though the upregulated transcripts in the quiescent state were orders of magnitude lower in abundance relative to active growth state transcripts, their protein levels increased. Second, upon encountering favorable conditions, protein levels of active growth-associated genes increased within minutes, well before their upregulation at the transcriptional level (23). Therefore, we hypothesized that genes encoding proteins of the translation system (e.g., RPs, translation factors, RNases, and RNA-modifying enzymes) are regulated to produce a diverse population of ribosomes that selectively translate physiological state-specific transcripts relevant for a given environmental condition.

To test this hypothesis, we performed sequential window acquisition of all theoretical mass spectra (SWATH-MS) proteomics in concert with RNA sequencing and ribosome profiling in the halophilic archaeon *H. salinarum*. This analysis revealed preferential translation of upregulated and low abundance transcripts. In an attempt to uncover the underlying mechanism for this phenomenon, we discovered that transcriptional, translational, and posttranslational regulatory mechanisms act on RP genes across the growth phase to effect conditional changes in their abundance and stoichiometric association with the assembled ribosome. We further analyzed previously developed environment and gene regulatory influence network (EGRIN) models (25) for signatures of heterogeneity in the whole translation system. We discovered that transcriptional regulation of RPs is fractured into multiple mostly discrete but partially overlapping conditionally coregulated gene modules (corems). Subsets of translation system genes associated with tRNA charging (aminoacyl-tRNA synthetases), translation factors (e.g., initiation, elongation, release, and recycling factors), and RNA transcription and processing (e.g., RNA polymerase, RNases, and RNA modification enzymes) are split among conditionally coregulated corems. Notably, we observed similar modular regulation of ribosomal proteins in E. coli and yeast. This modular and environment-specific regulation of ribosomal proteins might have emerged to favor evolvability (26) and functional specialization of ribosomes (27). In sum, our data support the hypothesis that environment-specific ribosome composition and coupled transcription-translation in prokaryotes selectively bias translation of low abundance and upregulated transcripts to produce proteins needed for the environment-relevant physiological state.

## RESULTS

### Interplay of transcriptional and translational regulation across growth phase-associated physiological state transitions.

**FIG 1:**
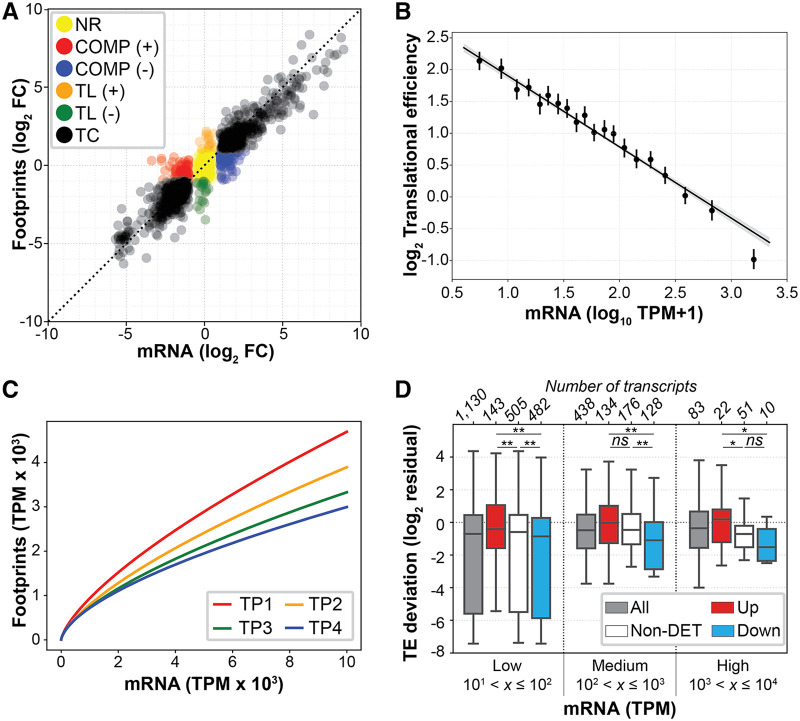
Coupled transcription-translation regulation across growth phase. (A) Scatterplot of relative changes in transcript abundance (log_2_ FC mRNA) and ribosomal footprints (log_2_ FC footprints) in stationary phase (TP4) with respect to early exponential phase (TP1). Black dots (TC; *n *= 875) represent genes regulated at the transcriptional level only. Red (*n *= 79) and blue (*n *= 110) dots represent genes under positive (+) and negative (-) compensatory mechanisms, respectively (COMP). Orange (*n *= 15) and green (*n *= 24) dots represent genes translationally regulated only (TL). Yellow dots represent genes that are not transcriptionally or translationally regulated (NR; *n *= 304). (B) Linear regression of transcript abundance (*x* axis; log_10_ TPM + 1) and TE (*y* axis; log_2_ Ribo-seq/RNA-seq ratio). Slope, *a* = −1.10; correlation coefficient *R* = −0.52; *P* < 10^−132^. The gray area represents the 95% confidence interval. (C) Regression analysis of predicted ribosomal footprints from transcript expression at different growth phases. (D) Deviation distributions from the expected TE given expression (*y* axis) in the context of transcriptional regulation across growth phase (stationary versus early exponential; TP4 versus TP1). Transcriptionally upregulated genes are shown in red, downregulated genes in blue, nondifferentially expressed (non-DET) genes in white, and all genes in gray. The horizontal axis shows expression levels (*x*): low, 10 < *x *≤ 100; medium, 100 < *x *≤ 1,000; high, 1,000 < *x *≤ 10,000. The number of transcripts of each boxplot (*n*) is shown at the top. Asterisks indicate significance: *ns*, nonsignificant; *, *P* < 0.05; **, *P* < 0.01.

When H. salinarum cells transition from lag to stationary growth phase, they travel across multiple regulatory configurations or states involving gene expression changes in more than two thirds of all genes in the genome (24). In order to investigate the interplay of transcriptional and translational regulation at the whole-genome scale for the growth-associated physiological state transition in *H. salinarum*, we quantified for each transcript the relative change in its abundance and ribosomal footprints across all phases of growth in batch culture. Specifically, we probed four time points representative of different growth phases, i.e., early exponential (time point 1 [TP1]), mid-exponential (TP2), late exponential (TP3), and stationary (TP4). For each sampled time point, we quantified the transcriptome state using RNA sequencing (RNA-seq) and ribosome translational activity using ribosome profiling (Ribo-seq) (see Materials and Methods for details). We used a filter on magnitude (absolute log_2_ fold change [FC] > 1) and significance (DESeq2-adjusted *P* < 0.05) to assess both transcript abundance and ribosomal footprint changes across growth phase (for a detailed list of transcript quantification, see Table S1, tabs 1 to 12, in the supplemental material). Of the total set of 2,663 annotated genes in *H. salinarum*, 304 were not regulated at the transcriptional or translational level (yellow dots in Fig. 1A). Among the 1,103 genes that were subjected to regulation, changes in ribosomal footprints per transcript for 875 genes (79%) are proportional to changes in transcript abundance (black dots in Fig. 1A). Among this set, upregulated genes were significantly enriched with functions associated with gas vesicle organization (GO:0031412) and cell motility (GO:0048870), protein phosphorylation (GO:0006468), the iron-sulfur cluster (GO:0016226), reactive oxygen species metabolism (GO:0072593), and response to stress (GO:0006950). Functions enriched within downregulated genes included nitrogen and phosphorous metabolism (GO:0006807 and GO:0006793), carbohydrate catabolism (GO:0016052), pyruvate biosynthesis (GO:0042866) and energy production via proton-exporting ATPase activity (GO:0036442).

10.1128/mSystems.00329-20.10 TABLE S1 (Tabs 1 and 2) Transcript and ribosomal footprint abundance quantification processed with Trimmomatic and kallisto. (Tabs 3 and 4) Quantification of transcript and ribosomal footprint relative changes in stationary phase (TP4) with respect to early exponential (TP1). Raw sequence data were processed with Trimmomatic, STAR, HTSeq-count, and DESeq2 pipeline. (Tabs 5 to 12) Quantification of transcriptional and translationally regulated genes across growth phase. Color-coded groups are shown in Fig. 1A in the main text, along with their fold change and significance values computed by DESeq2. (Tabs 13 to 19) We used the Bioconductor package topGo to discover significantly enriched GO terms in different transcript sets of interest. We trimmed out results with less than two transcripts per GO term. (Tab 20) arCOG-based functional enrichment. (Tabs 21 and 22) SWATH-MS quantification of RPs and their stoichiometry. (Tab 23) Transcript fold changes per groups as in Fig. 3A and B in the main article. (Tab 24) Operon memberships. We mined our HaloEGRIN2.0 model for gene regulatory elements (GREs) upstream of the genes of the two groups. We computed the distribution of GREs 100 bp upstream of each gene. We found a clear bias in GRE presence in the two groups—while GRE #9 and GRE #28 dominate among group A genes, these two GREs are not present at all in group B genes, which appear to be regulated by GRE #7 (Tab 25). Importantly, GRE #7 is substantially different from both GRE #9 and #28 (Fig. S6). Consequently, we propose that the expression difference between the two groups of genes can be explained by their promotor architecture differences. (Tab 25) Gene regulatory elements in ribosomal protein gene groups. (Tab 26) Class membership. (Tab 27) Ribosomal protein names and corem membership information. (Tab 28) Gene and protein name and identifier (ID) correspondence. Download Table S1, XLSX file, 1.5 MB. Copyright © 2020 López García de Lomana et al. 2020 López García de Lomana et al. This content is distributed under the terms of the Creative Commons Attribution 4.0 International license.

Interestingly, ribosomal footprint changes in 228 genes (21%) could not be explained by transcript level change, i.e., ribosomal footprints were greater or less than expected relative to change in transcript level. This observation is in agreement with an earlier report which showed that more than 20% of the *H. salinarum* transcriptome exhibits nonaverage translational efficiency (TE) (quantified as the log_2_ ratio of ribosomal footprints over transcript abundance [28]) in stationary versus exponential phase (29). Among this set of translationally regulated genes, 189 genes displayed compensatory mechanisms (30). First, a set of 110 genes were upregulated at the transcriptional level, which was not reflected in a corresponding increase in their ribosomal footprints (blue dots in Fig. 1A). This set of genes are involved in phospholipid and polysaccharide metabolism (GO:0006644 and GO:0000271), transcription initiation (GO:0006352), and response to stimulus (GO:0050896). Inversely, 79 genes had a significant decrease in transcript levels, although their ribosomal loading remained constant (red dots in Fig. 1A). This set of genes encode functions of DNA replication and repair (GO:0006261 and GO:0006284), modified amino acid biosynthesis (GO:0042398), and electron transfer activity (GO:0009055).

Orthogonally, 39 genes changed only at the translational level, i.e., while their mRNA levels did not change significantly, their ribosomal footprint abundances did (orange and green dots in Fig. 1A). Among the translationally upregulated genes, we noted *VNG2625C*, which encodes a PrsW family protease, and *nrnA*, which encodes a protein that is a bifunctional oligoribonuclease/PAP phosphatase which regulates degradation of nanoRNAs in bacteria (31). The translationally downregulated genes were enriched in redox energy metabolism functions, specifically components of the electron transport chain, including NADH-dependent flavin oxidoreductase gene *VNG0933G*, cytochrome *c* biogenesis protein gene *VNG0150H*, and nicotinate-nucleotide pyrophosphorylase gene *VNG1884G*, in addition to the PadR family transcriptional repressor *VNG7102*. A detailed list of all enriched functions is provided in Table S1, tabs 13 to 20; additionally, a visualization summary is provided as Fig. S1B to H in the supplemental material.

10.1128/mSystems.00329-20.1 FIG S1 (A) Quality control for RNase I treatment. We tested the effect of RNase I on both sucrose cushion (left) and spin column (right) isolation samples. RNase I treatment generated bands which are consistent with the expected size of ribosome footprints (∼30 nucleotides [nt]). Notably, these bands are absent without RNase treatment. Intense high-molecular-weight bands correspond to rRNAs. (B to H) Summarized GO enrichments by REVIGO from transcriptomics analysis: (B) transcriptionally upregulated; (C) transcriptionally downregulated; (D) translationally upregulated; (E) translationally downregulated; (F) compensatory, positive; (G) compensatory, negative; (H) not changing. Download FIG S1, TIF file, 2.6 MB. Copyright © 2020 López García de Lomana et al. 2020 López García de Lomana et al. This content is distributed under the terms of the Creative Commons Attribution 4.0 International license.

These observations are consistent with the known physiological shift of *H. salinarum* when it transitions from early exponential to stationary phase of growth (24). In summary, while 79% of all regulated genes have consistent changes at the mRNA level and ribosomal footprints (*n *= 875), there is significant evidence for interplay of transcriptional and translational regulation for at least 228 genes (21%).

### Selective translation of low abundance and upregulated transcripts.

Next, we investigated the potential for ribosomes to regulate physiological transitions through different phases of active growth and into a quiescent state in stationary phase. In particular, we asked whether and how the translation machinery selectively translates low abundance and upregulated mRNAs that are required for homeostasis in each growth phase. By comparing transcript abundance to TE, we discovered that TE negatively correlates with mRNA expression (slope = −1.10; *R* = −0.52; *P* < 10^−132^; Fig. 1B), irrespective of transcript length or transcript half-life (data obtained from reference 32; see Fig. S2 for more details). This negative relationship exists at all physiological states across growth phase, becoming stronger in stationary phase (Fig. 1C). This finding suggests that ribosomes associate more efficiently with low abundance transcripts. This finding was further supported by the observation that the 15 transcripts that are exclusively upregulated at the translational level [TL (+) in Fig. 1A] are low in abundance (Fig. S3). Furthermore, we discovered across all levels of expression that transcriptionally upregulated genes were associated with higher TE with respect to transcriptionally downregulated genes (Mann-Whitney U test, *P* < 0.05; Fig. 1D). This observation supports the notion that transcription and translation are coupled in archaea and is consistent with our discovery of selective translation of upregulated transcripts across growth-associated physiological transitions in *H. salinarum*. Through the analysis of corresponding changes in transcript levels and ribosome occupancy in E. coli (33), we have discovered compelling evidence that bacteria also preferentially translate low abundance transcripts. However, we did not see evidence for this phenomenon in yeast (34), suggesting that this phenomenon might be exclusive to prokaryotes (Fig. S4).

10.1128/mSystems.00329-20.2 FIG S2 TE association with other transcript features. (A) Scatterplot showing the relationship between TE and expression. We analyzed separately transcripts with ribosomal footprints (Ribo-seq > 0; black dots and line) from transcripts with no footprints (Ribo-seq = 0; tan line). (B) Regression plot using 10 bins for transcripts with footprints (Ribo-seq > 0). (C) Regression plot using five bins for low abundance transcripts (lower than the first quartile [Q1]). (D to F) As in panels A to C for transcript length and TE. (G to I) As in panels A to C for transcript half-life and TE. (J to L) As in panels A to C for expression and half-life. A similar trend between mRNA abundance and transcript half-life has been reported in E. coli by Bernstein et al. (53)—as the authors suggested, transcription initiation may be the dominant factor in determining mRNA steady-state levels in the cell, while mRNA decay would serve as a mechanism to respond rapidly to environmental changes. Download FIG S2, TIF file, 2.2 MB. Copyright © 2020 López García de Lomana et al. 2020 López García de Lomana et al. This content is distributed under the terms of the Creative Commons Attribution 4.0 International license.

10.1128/mSystems.00329-20.3 FIG S3 Genes upregulated exclusively at the translational level have low abundance. The solid black histogram represents expression distribution of all genes. Colored dashed histograms represent distribution of mean expression for 1 × 10^6^ randomly selected sets of size equal to the group of interest. Solid vertical lines represent mean expression of the group of interest in early exponential phase (TP1). (A) For the two gene sets translationally regulated exclusively [TL (+), *n *= 15; TL (-), *n *= 24], only upregulated genes are expressed at a lower abundance than expected by chance [TL (+), *P = *0.0022; TL (-), *P = *0.32]. (B) Transcriptionally regulated genes have a consistent pattern of expression change, i.e., upregulated genes have lower abundance than expected by chance [COMP (-), *P* < 1 × 10^−6^, *n *= 110; TC (+), *P* = <1 × 10^−6^, *n *= 442] and vice versa [COMP (+), *P* = 0.0324, *n *= 79; TC (-), *P < *1 × 10^−6^, *n *= 433]. (C) Expression of nonregulated transcripts does not deviate from expected by chance (NR, *P* = 0.0991, *n *= 304). Download FIG S3, TIF file, 2.8 MB. Copyright © 2020 López García de Lomana et al. 2020 López García de Lomana et al. This content is distributed under the terms of the Creative Commons Attribution 4.0 International license.

10.1128/mSystems.00329-20.4 FIG S4 TE association with expression abundance in other species. Negative association of TE and expression abundance observed in *H. salinarum* (A) holds to a certain extent in E. coli (B) but not in S. cerevisiae (C). Download FIG S4, TIF file, 1.6 MB. Copyright © 2020 López García de Lomana et al. 2020 López García de Lomana et al. This content is distributed under the terms of the Creative Commons Attribution 4.0 International license.

### RP abundance and composition within ribosomes across growth phase-associated physiological states.

**FIG 2:**
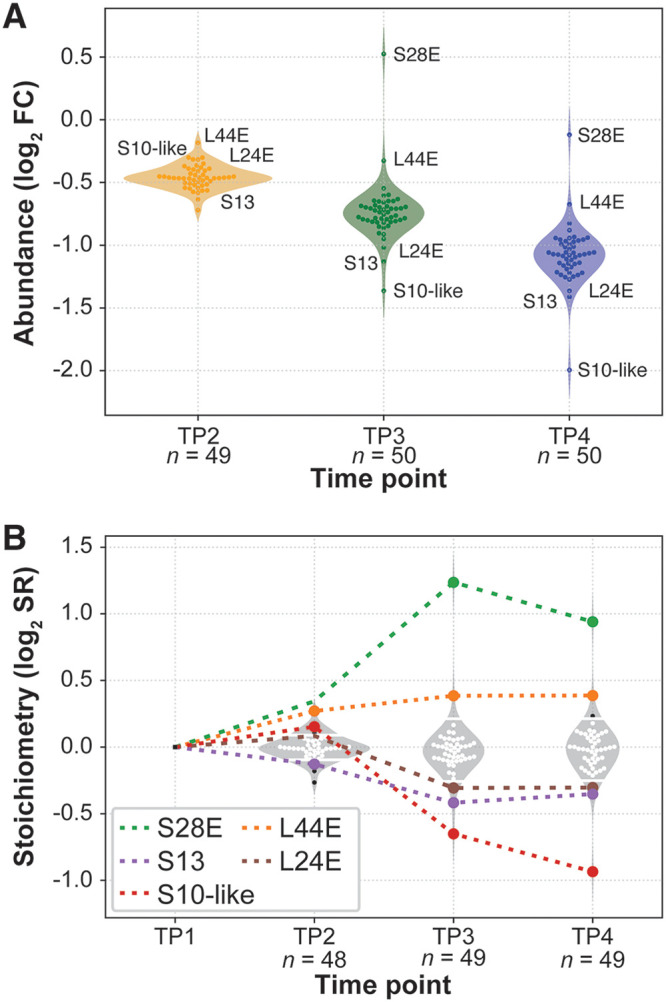
Ribosome abundance and composition shifts across growth-related physiological states. (A) Relative RP abundance changes (log_2_ FC) with respect to early exponential phase (TP1). Protein detection ranged from *n *= 49 to *n *= 50 RPs across samples. (B) Ribosome composition (log_2_ RP stoichiometry ratio) changes across growth phase. Small white dots represent RP stoichiometry values, and small black dots represent values outside the 95% confidence interval (95% CI). Large colored dots highlight RPs with two or more deviation events outside the 95% CI threshold. Dashed colored lines assist picturing the trend across time. White horizontal bars inside violin plots indicate 95% CIs.

The preferential translation of low abundance and upregulated transcripts, as well as translational regulation of 228 genes (21% of all regulated genes), motivated further investigation into a mechanistic explanation for these phenomena. It has been demonstrated in other organisms that differential RP stoichiometry plays a major role in driving physiological modulation during cell growth (9). Therefore, we assessed protein abundance and composition of assembled ribosomes using quantitative proteomics, specifically sequential window acquisition of all theoretical mass spectra (SWATH-TM; see Materials and Methods and Table S1, tabs 21 and 22, for details). We observed a progressive decrease of RP abundance as growth phase advanced toward stationary phase, when a median log_2_ FC = −1.04 indicates a general repression of translation in the cell (Fig. 2A). Notably, relative abundance changes in some RPs deviated across time from the overall trend, suggesting that ribosomes with distinct RP compositions were active in each of the different growth-associated physiological states.

As expected, ribosome composition was generally conserved but had few notable exceptions. Relative stoichiometry ratio (SR) of five RPs consistently deviated from the general trend across multiple time points, with most significant stoichiometric deviations of approximately twofold increase and decrease in S28E and S10-like proteins, respectively (Fig. 2B). Within statistical significance, we observed higher ribosomal associations of S28E (log_2_ SR = 0.94) and L44E (log_2_ SR = 0.39). Both RPs with higher stoichiometry belong to a three-gene operon together with *ndk* (which encodes a nucleoside diphosphate kinase), a conserved gene across all domains of life with pleiotropic effects in a wide range of functions (35). The RP stoichiometry of S28E and L44E increased despite significant downregulation of the operon at the transcriptional level (5,483 to 342 transcripts per million [TPM]; log_2_ FC = −4.00; *P* < 1 × 10^−7^). The differential association of the two RPs in the ribosome has implications on regulation of translation, given that S28E is located at the mRNA exit site in eukaryotes (36) and L44E is a conserved component of the E-tRNA site (37) of the eukaryotic and archaeal ribosome where it interacts with initiation factor eEF3 (38). Similarly, ribosomes in stationary phase have lower stoichiometry for three RPs: S10-like, S13, and L24E with ratios of log_2_ SR = −0.94, log_2_ SR = −0.35, and log_2_ SR = −0.30, respectively. At least two of these RPs have also been critically implicated in regulatory functions: while not much is known about S10-like protein, S13 is a conserved protein across archaea, bacteria, and eukaryotes that controls mRNA-tRNA complex translocation (39–41), and L24E binds to translation initiation factor IF6, which is conserved in archaea and eukaryotes (42). Thus, protein composition and abundance of the pool of assembled ribosomes progressively change across different stages of growth. Importantly, five RPs occur in different stoichiometry within assembled ribosomes in stationary phase compared to early exponential phase. We predict that these five RPs play an important role in growth phase-dependent regulation of protein synthesis in *H. salinarum*.

### Transcriptional and translational regulation of RP genes.

**FIG 3:**
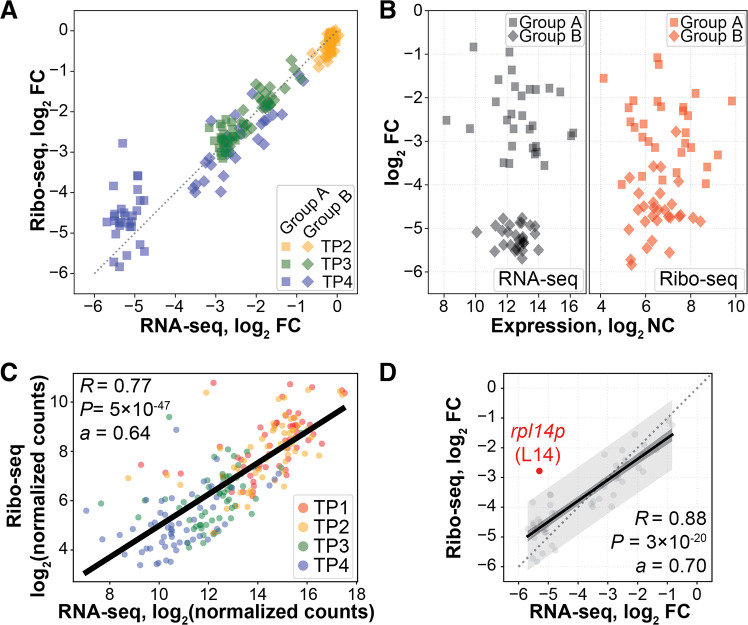
RP genes are transcriptionally and translationally regulated. (A) Scatterplot of relative abundance changes with respect to TP1. Each dot represents median log_2_ FC over three biological replicates for a given RP gene. Colors map to time points. (B) Scatterplot of expression and relative change of RP gene abundance (RNA-seq; left) and ribosomal footprints (Ribo-seq; right) at time point TP4 compared to TP1. (C) Scatterplot of absolute transcript and footprint abundances measured across growth phase. Each dot represents the median log_2_ normalized counts over three biological replicates for a given RP transcript. Colors map to time points. *R*, correlation coefficient; *a*, slope. (D) Scatterplot and regression model of RP transcript and footprint abundance changes in time point TP4 compared to TP1. The black line represents regression model, the dark gray area represents regression confidence interval, and the light gray area represents the regression prediction interval. The red dot highlights *rpl14p* outside the prediction interval. *R*, correlation coefficient; *a*, slope.

We investigated whether transcriptional and translational regulatory mechanisms account for the differential RP stoichiometry we observed. Interestingly, changes in RP transcript levels appeared to be mediated by at least two distinct processes in stationary phase with one group of 29 transcripts (group A) with median stationary phase downregulation of log_2_ FC = −2.04 and a second group of 29 transcripts (group B) with log_2_ FC = −4.23 (blue dots in Fig. 3A). While multiple mechanisms contribute to transcript level changes (new transcription, RNA stability, targeted degradation, etc.), this bipartite transcriptional regulatory program is most likely an outcome of distinct transcriptional regulation of RP genes in group A and group B and not operon structure (Table S1, tabs 23 to 25, and Fig. S5 and S6).

10.1128/mSystems.00329-20.5 FIG S5 Operon structure of ribosomal protein genes and their relative expression changes across growth phase. Operon names are marked as subpanel titles. Different colors indicate the genes; black indicates nonribosomal protein genes. Gray arrows indicate transcription initiation. FC, fold change; TP, time point. More details in Table S1 Tab 24. Download FIG S5, TIF file, 2.8 MB. Copyright © 2020 López García de Lomana et al. 2020 López García de Lomana et al. This content is distributed under the terms of the Creative Commons Attribution 4.0 International license.

10.1128/mSystems.00329-20.6 FIG S6 Motif logos for the GREs of interest. Download FIG S6, TIF file, 2.7 MB. Copyright © 2020 López García de Lomana et al. 2020 López García de Lomana et al. This content is distributed under the terms of the Creative Commons Attribution 4.0 International license.

To understand the implications of the bipartite transcriptional regulation, we analyzed transcript abundances, ribosomal footprints, and protein levels of RPs across different stages of growth. The bipartite grouping was apparent only in relative expression and not at the level of absolute expression or ribosomal footprints (Fig. 3B). There was significant correlation between change in abundance of RP transcripts and ribosomal footprints (slope = 0.64; *R *= 0.77; *P* < 10^−46^; Fig. 3C). RP transcript abundance decreased as growth phase advanced—from 4,719 median normalized counts in early exponential (TP1) to 366 in stationary phase (TP4). Ribosomal footprints followed a similar trend, from 1,761 in TP1 to 217 in TP4. In fact, an ordinary least-squares regression model demonstrated that all footprint changes were within the expected prediction interval based on transcript level changes (slope = 0.70; *R *= 0.88; *P* < 10^−20^; Fig. 3D). For only one instance, *rpl14p*, we observed ribosomal footprints significantly more abundant than expected by chance. While the *rpl14p* transcript level was downregulated, its ribosomal footprints did not change proportionally. Given *rpl14p* transcript levels and downregulation, we would expect a 26-fold downregulation (log_2_ FC = −4.70) of its footprint levels. However, *rpl14p* exhibits only a sevenfold downregulation (log_2_ FC = −2.78). Unexpectedly, we did not observe a significant deviation of L14 at the level of protein abundance and ribosomal stoichiometry, suggesting that deviation of changes in ribosomal footprints for *rpl14p* is likely a false-positive result, especially given that it is in the middle of a large 20-gene operon. Consistently for all RPs upon transition to stationary phase, downregulation was much more pronounced at the transcriptional (log_2_ FC = −3.68) and ribosomal footprint (log_2_ FC = −3.02) levels, relative to protein level (log_2_ FC = −1.04), possibly reflecting the well-known fact that proteins are more stable than mRNAs. Taken together, these observations demonstrate that relative changes in transcript abundance and TE of RP genes do not manifest at the protein level, thus maintaining the overall conserved stoichiometry of RPs within ribosomes.

### Modular programs govern conditional regulation of RP transcription.

Given the extensive growth phase-dependent mRNA changes in RP genes, we explored a whole-genome gene regulatory network of *H. salinarum* EGRIN model to determine whether regulation of translation system genes was governed by context-specific transcriptional programs. In brief, the EGRIN model of *H. salinarum* was constructed in two steps (43). First, the cMonkey algorithm (44, 45) was used to discover biclusters, which are sets of conditionally coregulated genes that share conserved gene regulatory elements (GREs) in their promoters. Second, regulators for each corem were inferred using Inferelator (46, 47), which explains and predicts relative changes in expression levels of genes within each bicluster as a weighted sum of corresponding or preceding changes in transcriptional and environmental factors. We further developed an EGRIN2 model (25) which consists of an ensemble of EGRIN models—each constructed with a different data subset from a compendium of 1,495 transcriptome profiles from diverse environmental conditions. EGRIN2 models high-confidence associations among genes, environments, GREs, and regulators based on frequency of their cooccurrence within biclusters across all EGRIN models, resulting in corems. Corems are modular entities in EGRIN2 that capture with high confidence specific environmental context and regulatory mechanisms for coregulation of genes, and therefore, EGRIN2 represents an ideal framework to investigate condition-specific regulation of the translation machinery.

**FIG 4:**
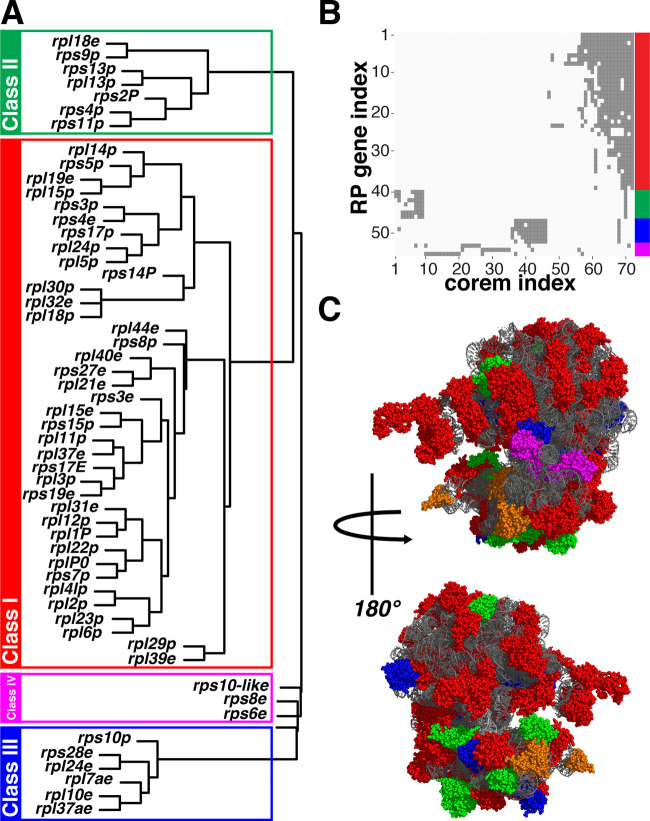
RP genes are organized into distinct functional coregulated classes. (A) Bootstrapped hierarchical clustering of RP genes based on corem membership identified four classes, including three principal classes plus three outlier genes, joined together as class IV. Classes are boxed and colored with red, green, and blue, and outlier genes in magenta. (B) RP genes are depicted on the *y* axis versus corems on the *x* axis. Dark gray squares indicate the presence of a particular gene in a given corem. The RP genes are arranged and colored by class on the right side. Corems comprise both neighboring genes in operons but also distal genes. (C) RPs are depicted in the ribosomal 3D structure following the color scheme of the functional classes as shown in panel A. Functional classes of RPs do not follow a restricted pattern of physical interactions, indicative of functional specialization of the ribosome due to coregulation in different environments, rather than coexpression derived from physical interactions at the protein level. Gray sections represent rRNA molecules. Subunits excluded (S12P, S19E, S24E, and S27AE) from the clustering analysis because they were not present in any corem are depicted in orange.

With the exception of four RP genes (*rps19e*, *rps27ae*, *rps24e,* and *rps12P*) that were not grouped into any corem, 54 out of 58 annotated RP genes segregated into four classes based on applying hierarchical clustering on membership to 72 identified corems (Fig. 4A). These classes (corem membership information is detailed in Table S1, tabs 26 to 28), which we have labeled “class I-IV,” were also somewhat distinguished by operon and genome architecture, as would be expected from the inclusion of sequence features in the biclustering algorithm. For example, a large operon encoding 22 RPs, the RNase P protein subunit, the SecY translocation protein, and a putative RNA methyltransferase, was fully represented in two corems. In all other corems, this operon was fragmented, meaning that subsets of genes within the operon were conditionally split and differentially coregulated as transcript isoforms (48). All corems containing genes of this operon included different combinations of an additional 16 RP genes scattered throughout the genome (Fig. 4B). Further, these four classes extended to other translational proteins (Fig. S7).

10.1128/mSystems.00329-20.7 FIG S7 Ribosomal proteins are coregulated within the context of translation and transcription systems. (A) Overall interaction of ribosomal protein genes with other genes of the translation and transcription systems. Genes are depicted as hexagons and modules as diamonds. Colors represent different functional categories of genes as follows: green, ribosomal proteins; blue, translation factors; red, RNA polymerase; magenta, RNase/RNA-binding proteins. Subnetworks of ribosomal protein classes as shown in Fig. 4 in the main text are magnified in panels B to E. (B) In addition to ribosomal proteins, class I includes 4 translation factors (Eif2a, Eif5a, Ef1b, and Rli), 2 RNA polymerase subunits (RpoL and RpoF), 4 RNase/RBP proteins (Snp, RNase P, Nop10p, and VNG1688C), 2 aminoacyl-tRNA synthetases (ValS and ProS), GrpE and SecY. (C) Class II of ribosomal proteins includes 3 RNA polymerase subunits (RpoK, RpoN, and Rpb3) and one RNase (Eno). (D) Class III includes one RNA polymerase subunit (RpoP) and one translation factor (Eef1a). (E) S6E is coregulated with translation factor EEF2 and RNase J (VNG1149Cm). Two RNA polymerase subunits (RpoE’/E’’), translation factors Eif2b and Eif2g, and putative ribosome biogenesis factor (Fcf) are coregulated and share modules with the other classes and S6E. Download FIG S7, TIF file, 2.7 MB. Copyright © 2020 López García de Lomana et al. 2020 López García de Lomana et al. This content is distributed under the terms of the Creative Commons Attribution 4.0 International license.

In the largest group, class I, 38 RP genes—12 belonging to the small subunit and 26 to the large subunit—are broadly coregulated across 26 corems, albeit with substantial differences among the individual corems. Class II, consisting of seven RP genes (five small subunit and two large subunit) was coregulated in three corems, fractured in seven more corems, and altogether coregulated with additional genes. In the genome, the seven RP genes are physically located in two consecutive operons also containing three RNA polymerase subunits. The functional coherence of this class is further emphasized by six out of the seven genes (*rps2P*, *rps4p*, *rps9p*, *rps11p*, *rps13p*, and *rpl13p*) being universal RP genes, with the lone exception of *rpl18e*. Class III consists of six RP genes (two small, four large subunits) across 12 corems, seven of which contained all six RP genes. Three of the RP genes are in an operon, while the other three are separated from the operon and each other on the chromosome. In contrast to class II, five RP genes (*rps28e*, *rpl7ae*, *rpl37ae*, *rpl10e*, and *rpl24e*) are specific to archaea and eukaryotes, with the lone exception being *rps10p*. Classes II and III are each coregulated with class I in single, discrete corems, further suggesting modular regulation of the ribosome as a whole. Three of the remaining RP genes (*rps6e*, *rps8e*, and *rps10*-like) show sparse association with each other and previous classes and were associated together as class IV. Two genes from the S10 ribosomal family, *rps10* and *rps10-like*, encode two ostensibly similar RPs that are divergently regulated. Further, *rps6e* encodes an RP that is located near the A-site of the ribosome, where it can interact with mRNA structures to regulate translation (Fig. 4C). Together, these findings suggest that specific conditional regulation of these proteins may lead to distinct ribosomes with functional specialization.

### Evidence and mechanisms of condition-specific regulation of RPs.

**FIG 5:**
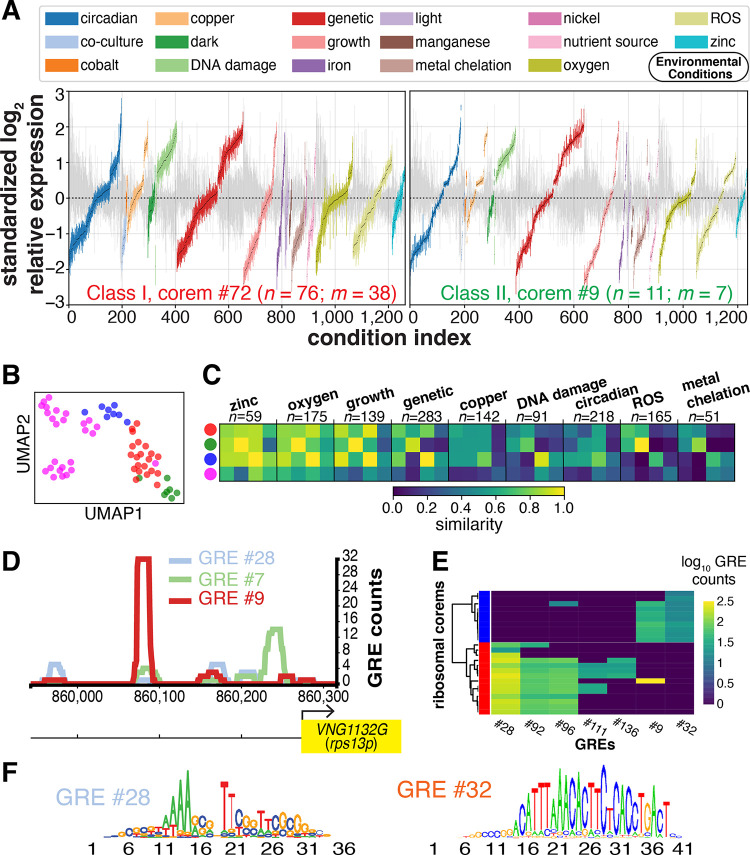
RP genes are coregulated in a condition-specific manner. (A) Expression distribution for two representative examples of the 72 ribosomal corems identified. Vertical bars represent interquartile range (third quartile [Q_3_] to first quartile [Q_1]_) of expression across genes. *n* indicates the number of genes in each corem, whereas *m* refers to the subset that encode RPs. Colors correspond to different environmental conditions (see key). Conditions are ranked based on median expression. Gray background bars correspond to average expression distribution of 10,000 permutations of randomly selected gene sets of the same size as the corem. Note the larger interquartile range of background distributions. (B) Corem similarity based on gene expression. We used uniform manifold approximation and projection (UMAP [77]) to visualize 62 ribosomal corems in a two-dimensional space from an original high-dimensional space of 1,495 environmental conditions. Each dot represents a corem. Color maps to functional classes. (C) Similarity matrix between ribosomal corem classes broken into nine different broad condition categories (*n*, number of experimental conditions). Similarity values across diagonals may not reach one, as we defined class similarity as the proportion of class corems with no significant expression differences. (D) Promoter architecture of *rps13p* (*VNG1132G*) deciphered by MAST (78) alignments of *cis*-regulatory motifs from each GRE, in this case GRE #7, #9, and #28 discovered by MEME (79) in gene promoters of EGRIN biclusters that include *rps13p*. The heights of the histograms are proportional to the frequency of GRE alignments to the *VNG1132G* promoter. (E) Hierarchical clustering of corems enriched in RP genes based on relative importance of each GRE (log_10_ GRE counts) coregulating corem members. Red and blue bars correspond to class I and III, respectively. (F) Motif sequence logos for two representative GREs.

Once we determined that RPs associate with corems containing genes with other cellular functions, we hypothesized that *H. salinarum* might conditionally regulate RP composition of ribosomes in different environmental conditions. We explored evidence for context-specific regulation of RPs by analyzing expression coherence of genes within and across corems of the four classes under a comprehensive set of environmental conditions. We observed that genes within corems of the same class are coregulated across many conditions (Fig. 5A), but there is variability across classes (Fig. 5B). Notably, we discovered that variability across corems from different classes are condition specific. Specifically, for each broad category of conditions—growth in batch culture, shifts between high and low oxygen, response to different metals, etc.—we computed expression similarity between ribosomal corem classes, defined as the proportion of corem comparisons that had no significant gene expression differences (Fig. 5C). We conclude that the degree of variability in coregulation of ribosomal genes across the four classes of corems is strongly dependent on environmental context.

We hypothesize that differential coregulation of RPs across environmental conditions should be apparent in distinct gene regulatory elements defining their promoter architecture. Specifically, we hypothesized that identified RP corem classes should have distinct GREs defining their promoter architecture. The EGRIN2 model predicts specific mechanisms for transcriptional regulation for every gene in the genome; for instance, it predicts that *rps13p* (*VNG1132G*) is regulated by at least three transcription factors via binding to three distinct ∼6- to 20-nucleotide GREs in the promoter of this gene (Fig. 5D and F). Furthermore, we demonstrated previously that EGRIN2 also accurately predicts which subset of GREs in the promoters of genes in corems are responsible for their environment-specific coregulation (25). Using this capability of EGRIN2, we investigated whether the observed variability in coexpression of RP genes across the corems of different classes was a consequence of different GRE composition. We performed hierarchical clustering on the composition of GREs implicated in coregulation of genes within corems enriched in RP genes (Fig. 5E). Distinct combinations of seven GREs are implicated in differential coregulation of ribosomal genes across the corems with at least one third of RP genes, supporting the hypothesis that distinct transcriptional regulatory mechanisms are responsible for the condition-specific variation in modular coregulation of RPs with one another and 614 other genes in the genome. We conclude from this analysis of the EGRIN2 model that variation in gene expression corresponds to environment-dependent modular regulation of ribosomal genes.

### Coregulation of translational complexes in E. coli and S. cerevisiae.

We investigated the generality of our findings from *H. salinarum* by analyzing the structure of conditional coregulation of RP genes in E. coli and S. cerevisiae. For these two organisms, the EGRIN models (25, 49) were mined in the same manner as for *H. salinarum*, and the resulting corems containing RPs, other translation factors, and the transcription apparatus were dissected. Analysis of the EGRIN models of E. coli and S. cerevisiae also showed that RP genes are organized into groups of corems, none of which encompasses all the genes in all circumstances. Rather, the set of RP genes is fractured into multiple mostly discrete, but partially overlapping, corems (Fig. S8). This indicates that ribosome composition regulation in all three organisms is less of a singular entity, and more of a mosaic patchwork. Additionally, subsets of translation system genes associated with tRNA charging and translation factors (including aminoacyl-tRNA synthetases, initiation, elongation, and release factors), as well as RNA handling (RNases and RNA modification enzymes) are split among the corems. This pattern was apparent in all three organisms tested, suggesting a conserved pattern in all domains of life. Since the conservation in regulatory network architecture stems from gene expression, in addition to sequence features, it is not simply a genome sequence comparison, but it represents a correspondence in active physiology of organisms in relation to particular environmental conditions. Thus, these findings are consistent with the idea that functional specialization drives modularity in biological systems. Such modular regulation has evolutionary implications, as modularity would facilitate the evolvability of the translational complexes (27).

10.1128/mSystems.00329-20.8 FIG S8 Coregulation of ribosomal proteins across the tree of life. Ribosomal proteins are depicted on the *y* axis versus corems on the *x* axis. Dark gray squares indicate the presence of a particular protein in a given corem in E. coli (A) and S. cerevisiae (B). Download FIG S8, TIF file, 2.8 MB. Copyright © 2020 López García de Lomana et al. 2020 López García de Lomana et al. This content is distributed under the terms of the Creative Commons Attribution 4.0 International license.

### Physical protein-protein interactions support coupled transcription-translation.

**FIG 6:**
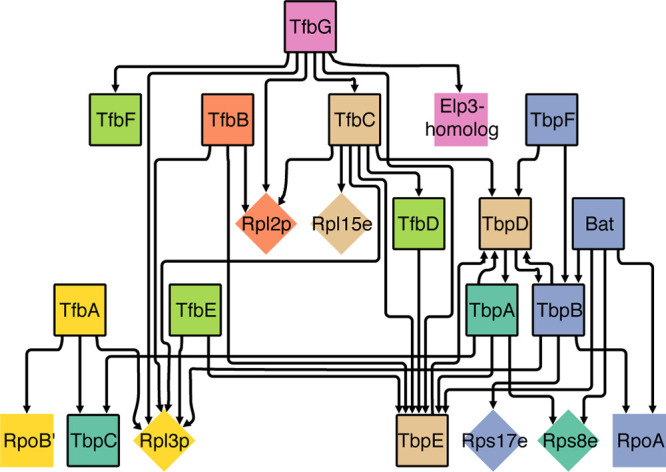
RPs physically interact with transcription complex components. Diamonds represent RPs; squares represent transcription complex components. Tagged proteins used as bait in the immunoprecipitation experiment are highlighted by a black border. Arrowheads link bait to coimmunoprecipitated proteins. We labeled each of the seven modules obtained by the Newman-Girvan clustering algorithm using a different color.

Physical interaction of the RNA polymerase (RNAP) with the 30S ribosomal subunit in prokaryotes (50–52) suggests that actively transcribed genes also actively recruit ribosomes for coupled translation. We investigated the evidence for a similar phenomenon in archaea by analyzing a protein-protein interaction map of *H. salinarum* constructed through immunoprecipitation of 14 protein A-tagged transcription complex components (22). In brief, 13 general transcription factors (GTFs)—six TATA-binding proteins (TBPs) and seven transcription factor B proteins (TFBs)—and bacterioopsin activator (Bat) were epitope tagged with protein A, and used as bait to immunoprecipitate *H. salinarum* transcriptional complexes in 14 independent experiments, performed in duplicate. We constructed a network of 128 proteins (as nodes) and 228 interactions, i.e., unidirectional edges from tagged baits to coimmunoprecipitated proteins that rendered seven modules (see Materials and Methods). This network presents 13 physical interactions between eight components of the transcriptional machinery and five ribosomal proteins (Fig. 6). Interestingly, while TFBs associated exclusively with the ribosome large subunit, TBPs preferentially interacted with the small ribosomal subunit. We further investigated whether coimmunoprecipitated RPs form an interacting interface in the ribosome. We explored their physical location in the ribosome three-dimensional (3D) structure. We found that while three of the five ribosomal proteins are scattered across the ribosome surface, L2 and L15E are particularly close to each other (Fig. S9). Nevertheless, functional implications of this observation require further investigation. Physical interactions between transcription and translation complexes have been previously implicated as the mechanism by which transcription and translation are coupled in prokaryotes, and here it gives a mechanistic hypothesis for why actively transcribed genes are preferentially translated. Moreover, this is consistent with the evidence we have provided that low abundance and upregulated transcripts are preferentially translated in both E. coli and *H. salinarum*, but not in yeast, because of physical separation of the two processes (Fig. S4).

10.1128/mSystems.00329-20.9 FIG S9 Physical location of the RPs coimmunoprecipitated with the transcription complex components. We used the same approach as in Fig. 4C in the main article to explore ribosome 3D structure. Small and large subunit proteins are represented in low-saturation blue and red, respectively; high-saturation colors are used to highlight specific RPs. (A) Standard view. S8E and L3P are highlighted in blue and red, respectively. (B) 180-degree view. L2, L15E, and S17E are highlighted. (C) 270-degree view. L2 and L15E are particularly close to each other. Download FIG S9, TIF file, 2.6 MB. Copyright © 2020 López García de Lomana et al. 2020 López García de Lomana et al. This content is distributed under the terms of the Creative Commons Attribution 4.0 International license.

## DISCUSSION

**FIG 7:**
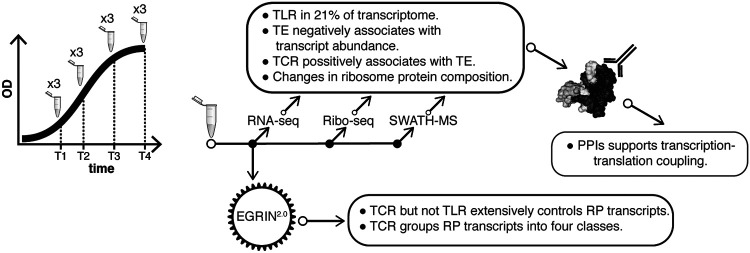
Synthesis figure. Growth phase *H. salinarum* transcriptome abundance, ribosomal footprints, and proteome quantification and analysis identified key points of translational regulation. TLR, translational regulation; TE, translational efficiency; TCR, transcriptional regulation; PPI, protein-protein interaction; OD, optical density; RP, ribosomal protein.

Here, we have demonstrated that preferential translation of low abundance and upregulated transcripts influences growth-associated physiological state shifts in *H. salinarum*. Characterizing the precise mechanism underlying this phenomenon has important implications for understanding how cells transition to a physiological state appropriate for a resource-limited environment (anoxia, nutrient starvation, etc.), which requires preferential and efficient translation of low abundance transcripts (Fig. 7).

Prior studies showed that highly expressed transcripts were upregulated and functionally required for processes such as aerobic respiration, ATP synthesis, tricarboxylic acid (TCA) cycle, transcription and translation, during oxic growth of *H. salinarum*—when the oxygen level dropped, the haloarchaeaon adopted a quiescent state and these transcripts were downregulated, but they continued to persist in high abundance, even though they were functionally irrelevant for anaerobic physiology (23). Importantly, protein levels of these downregulated transcripts also decreased during anoxia. In other words, even though these transcripts were present in high abundance, they were not actively translated in an anoxic environment. Further, *H. salinarum* reinitiated translation of these persistent highly abundant transcripts almost concurrently with increase in oxygen level, and well before their transcriptional upregulation (23). The current study proposes a mechanistic explanation for these classic observations.

Bernstein et al. (53) have demonstrated in E. coli that transcription initiation may be the dominant factor in determining mRNA steady-state levels in the cell, while mRNA decay might serve as a mechanism to respond rapidly to environmental changes. We hypothesize that most low abundance transcripts are transcribed constitutively at a low rate that is proportional to or less than their degradation rate, and therefore, they are associated with higher TE relative to highly abundant transcripts, which are expressed at a high level only in environments where their functions are relevant. Although outside the scope of this study, this hypothesis can be experimentally tested with GRO-seq and pulse-chase experiments (54, 55). Nonetheless, these observations are also consistent with both our finding that all transcriptionally upregulated genes have higher TE relative to downregulated transcripts, and the Schmid et al. finding that highly abundant transcripts are not translated in an unfavorable environment when they are transcriptionally downregulated (23). Alternate mechanisms can also explain why some upregulated transcripts are selectively translated in a growth phase-dependent manner. For examples, posttranslational modifications such as differential ubiquitination in eukaryotes can alter the stability of proteins to regulate ribosomal function such as by stalling assembled ribosomes and blocking access to initiation factors (56). While archaea do not possess the classical ubiquitination pathway, they do utilize hypusination to stop translation and growth via initiation factor aIF5A (57).

At a mechanistic level, the preferential translation of low abundance and upregulated transcript can be explained by the well-known phenomenon of coupled transcription and translation in prokaryotes (58, 59). At the molecular level, results presented in this study (Fig. 6) and previous reports for bacteria (50–52) and archaea (60) have demonstrated that the transcription machinery facilitates the recruitment of translation factors and the ribosome through physical protein-protein interactions. While this mechanism of transcription-translation coupling is pertinent to 875 *H. salinarum* genes that are regulated just at the transcriptional level, it is noteworthy that at least 228 genes (21% of all regulated genes) are subject to translational regulation during physiological state transitions. We observed two orthogonal modes of translational regulation: (i) changes in ribosomal footprints in fixed-abundance transcripts, and (ii) compensatory mechanisms, where ribosomal footprints remained unchanged in spite of transcript abundance changes. These translationally regulated genes encode a wide range of critical functions that include amino acid and lipid metabolism, DNA replication and repair, transcription regulation and energy homeostasis. This result has major implications on understanding physiological state transitions in archaea, as it has been already noted in human disease physiology (7, 61).

To that end, we discovered that regulation of translation systems is heterogeneous, and modular. Notably, these ribosomal modules (corems) also include 561 additional genes of diverse functions, including transcription, metabolism, signal transduction, and transmembrane transport, suggesting that regulation of components of the translation system is coordinated with the expression of diverse functions. This modular regulation of the translational machinery could provide a basis for specialization and adaptive evolution. There is extensive evidence that specialization leads to the emergence of modularity in biological systems, including in metabolic and transcriptional regulatory networks. There are at least two reasons why modularity facilitates the ability of an organism to generate adaptive heritable variation, i.e., evolvability. First, an organism can select variations inside one module, without perturbing other modules. Second, there is also evidence that modules can be repurposed or merged to generate novel functions (26, 62, 63). Despite the complex modular regulation of RP genes, we observed the expected and coordinated decrease in transcript and protein abundance of RPs when cells shifted to stationary phase. Concordantly, the majority of RPs in the ribosome-enriched fraction also decreased in a coherent manner, with very similar changes in relative abundance. These observations suggest that regulation of RP genes at the transcriptional and translational level has evolved to maintain stoichiometry of protein subunits within the assembled ribosome. However, ribosomal associations of five RPs significantly deviated upon transition to stationary phase, most likely driven by a posttranscriptional mechanism, and indicative of a pool of ribosomes with different RP stoichiometry that is responsible for variation in TE across physiological states (4, 18, 64). While further experimental validation is needed to demonstrate that ribosomes of distinct composition selectively translate specific sets of transcripts, our analysis of transcriptional regulatory networks of E. coli and yeast suggests that modular regulation and coordination of ribosomal proteins with other cellular functions are generalizable phenomena that underlie environment-specific adaptation and specialization of all organisms.

## MATERIALS AND METHODS

### Cell culture and sampling.

Wild-type Halobacterium salinarum NRC-1 was cultured in a liquid nutrient-rich complex medium (CM) (250 g/liter NaCl, 20 g/liter MgSO_4_·7H_2_O, 3 g/liter sodium citrate, 2 g/liter KCl, and 10 g/liter peptone [Oxoid, United Kingdom] made with distilled water). Cultures were inoculated to a starting optical density at 600 nm (OD_600_) of 0.02 with starter culture with an OD_600_ of 0.5 which was derived from a single colony. Cultures were grown in unbaffled flasks in which 40% of the flask volume is occupied by the culture. Cultures were grown at 37°C, shaken at 220 rpm, and illuminated at ∼20 μmol/m^2^/s in Innova9400 incubators (New Brunswick). Triplicate cultures were grown, and samples were harvested at four time points. The four time points were selected to represent the early exponential phase (OD_600_ of 0.2; 14.3 h), mid-exponential growth (OD_600_ of 0.5; 21.5 h), late exponential phase (OD_600_ of 0.8; 28.8 h), and stationary phase (40.8 h). The final time point was selected to be 12 h past the late exponential phase, since OD_600_ readings are not representative of cell growth in *H. salinarum* in stationary phase (24). At each time point, whole cells were collected by centrifugation for analysis by RNA sequencing, ribosome profiling, and mass spectrometry (MS) proteomics.

### RNA-seq and ribosome profiling analysis.

Cells were pelleted by centrifugation (8,000 × *g*, 2 min, 4°C), resuspended in a buffer containing 3.4 M KCl, 100 mM MgCl_2_, and 10 mM Tris-HCl at pH 7.4, sonicated at 4°C to lyse cells (amplitude 50%, pulse 30 s on and 15 s off, repeated 6 times), and centrifuged again at 14,000 × *g* for 10 min at 4°C to remove cell debris. Supernatants were collected and treated with RQ1 DNase (Promega), followed by centrifugation (14,000 × *g*, 10 min, 4°C). Ribosome-bound RNA was generated by treating the lysate with RNase I and quenching the reaction with Superase-In RNase inhibitor (see Fig. S1A in the supplemental material). Macromolecular complexes were collected by spin column isolation (MicroSpin S-400 HR; GE), elution was performed by centrifugation (600 × *g*, 2 min, room temperature), and samples were snap-frozen in liquid nitrogen and stored at –80°C. The elution sample was split into two aliquots, one for ribosome footprint sequencing and one for proteome analysis. For transcriptome sequencing, total RNA was collected from the cell lysate using TRIzol-chloroform extraction and elution with water. A total of 24 barcoded libraries were prepared for sequencing; 12 using the TruSeq Stranded mRNA HT library prep kit for mRNA, and 12 using the NEBNext Small RNA Library Prep Set from Illumina for the ribosome-bound fragments. Libraries were pooled, denatured, and diluted according to the NextSeq 500 protocol. Single-end sequencing of libraries was performed on the Illumina NextSeq 500 platform using two high-output flow cells with 75-bp read lengths. Adapter sequences were trimmed using Trimmomatic (65). Transcript abundance and ribosomal footprint quantification in the form of transcripts per million (TPM) was performed using kallisto (66) against a reference transcriptome of 2,665 open reading frames (ORFs). Differential gene expression analysis was performed using DESeq2 (67) (after HTSeq [68] and STAR [69]).

### Assembled ribosome protein analysis.

As described above, macromolecular complex isolation spin column (MicroSpin S-400 HR; GE), elution samples (enriched fractions) were obtained, snap-frozen in liquid nitrogen, and stored at –80°C. The protein content of the samples was determined by bicinchoninic acid assay (Thermo-Fisher). Proteins were reduced (5 mM dithiothreitol, 45 min, 37°C), alkylated (14 mM iodoacetamide, 30 min, room temperature, darkness), and digested with trypsin (1:50 enzyme/substrate ratio, 37°C, 16 h). Samples were desalted with tC18 SepPak cartridges (Waters). Samples were analyzed with a TripleTOF 5600+ system equipped with a Nanospray-III source (Sciex) and an Eksigent Ekspert nanoLC 425 with cHiPLC system in trap-elute mode (Sciex). Peptides were separated with a gradient from 3% to 33% 0.1% formic acid in acetonitrile (vol/vol) in 120 min. Data were collected in MS/MS^ALL^ SWATH acquisition mode using 100 variable acquisition windows. Data were analyzed with the OneOmics SWATH Proteomics Toolkit (Sciex) within the BaseSpace cloud computing environment (Illumina). An ion library was generated from *H. salinarum* grown to mid-exponential and stationary phase acquired in shotgun mode (information-dependent acquisition scanning of mass spectrometry performed in tandem [IDA-MS/MS]) with the TripleTOF 5600+ system. A confidence filter of ≥75% (statistically significant differentially expressed proteins) was applied to report protein expression changes.

### Gene ontology analysis and visualization.

Gene ontology (GO) annotations for each *H. salinarum* gene were obtained from MicrobesOnline (70). We used the Bioconductor package topGO (71) to discover significantly enriched GO terms in gene sets of interest. We used REVIGO (72) to summarize and visualize enriched GO terms.

### Cluster analysis.

Corems were identified based on an extensive pipeline that was previously published (25). To group corems by similarity of gene content, hierarchical agglomerative clustering of genes based on presence or absence in a corem was performed in R. The method used to create the distance matrix therefore was binary, and the clustering algorithm was average similarity. The clusters were bootstrapped using the package pvclust 10,000 times, and maximal clusters with >95% significant *P* values were selected, resulting in four robust classes. Genes that were not present in any corem were excluded from the analysis. In order to compare two given EGRIN2 corems, we computed both Spearman’s rank correlation coefficient (SRCC) and Kolmogorov-Smirnov test (KST) on their expression signatures over all conditions. If correlation was positive and significant (SRCC > 0.4 and *P* < 0.05) and KST was nonsignificant (*P* > 0.05), we considered that the corems had no significant expression differences.

### Structural modeling.

To obtain a visual sense of the RP classes, subunits were analyzed in PyMol. The Haloarcula marismortui large ribosomal subunit (PDB 4V9F) was aligned to the large ribosomal subunit of the archaeon Pyrococcus furiosus (PDB 4V6U). The P. furiosus large subunit was then hidden, while the small subunit was kept. rRNA structures were colored gray, and the RPs were colored according to their class or lack thereof.

### Protein-protein interaction network analysis.

Protein interaction data were retrieved from Supplementary Material Data Set 3 from Facciotti et al. (22). We removed duplicate entries. We retrieved protein annotation from NCBI Assembly (ASM680v1; RefSeq annotation), MicrobesOnline (73), and the Halobacterium salinarum NRC-1 SBEAMS database (https://baliga.systemsbiology.net/projects/halobacterium-species-nrc-1-genome). We used the Newman-Girvan algorithm (74) implemented in clusterMaker2 (75) for Cytoscape (76) version 3.7.2 to call network modules. To highlight interactions between general transcription factors and ribosome proteins, we hid all the nodes and edges not connected to them, and applied Cytoscape yFiles Hierarchic Layout. We minimally shifted the position of a few nodes to improve network legibility.

### Data availability.

RNA sequencing data have been deposited into NCBI SRA under BioProject number PRJNA413990. Mass spectrometry data are available in the PeptideAtlas data repository: http://www.peptideatlas.org/PASS/PASS01559. All code implementation, including sequence quantification and EGRIN model analyses, is available at the GitHub repository: https://github.com/adelomana/30sols.
